# Patient centered guidelines for the laboratory diagnosis of Gaucher disease type 1

**DOI:** 10.1186/s13023-022-02573-6

**Published:** 2022-12-21

**Authors:** A. Dardis, H. Michelakakis, P. Rozenfeld, K. Fumic, J. Wagner, E. Pavan, M. Fuller, S. Revel-Vilk, D. Hughes, T. Cox, J. Aerts, the International Working Group of Gaucher Disease (IWGGD)

**Affiliations:** 1 Regional Coordinator Centre for Rare Disease, University Hospital of Udine, P.Le Santa Maria Della Misericordia 15, 33100 Udine, Italy; 2 Department of Enzymology and Cellular Function, Institute of Child Health, Athens, Greece; 3 Departamento de Ciencias Biológicas, Facultad de Ciencias Exactas, Instituto de Estudios Inmunológicos Y Fisiopatológicos (IIFP), UNLP, CONICET, Asociado CIC PBA, La Plata, Argentina; 4 Department for Laboratory Diagnostics, University Hospital Centre Zagreb and School of Medicine, Zagreb, Croatia; 5 Department of Medical Biology and Genetics, Faculty of Medicine, J.J. Strossmayer University, Osijek, Croatia; 6 International Gaucher Alliance, Dursley, UK; 7 Genetics and Molecular Pathology, SA Pathology at Women’s and Children’s Hospital and Adelaide Medical School, University of Adelaide, Adelaide, SA 5005 Australia; 8 Gaucher Unit, Shaare Zedek Medical Center, Jerusalem, Israel; 9 Faculty of Medicine, Hebrew University, Jerusalem, Israel; 10 Lysosomal Storage Disorders Unit, Royal Free London NHS Foundation Trust and University College London, London, UK; 11 Department of Medicine, University of Cambridge, Cambridge, UK; 12 Department of Medical Biochemistry, Leiden Institute of Chemistry, Leiden, The Netherlands

**Keywords:** Gaucher disease, Biomarkers, Enzyme activity, Genetic testing

## Abstract

Gaucher disease (GD) is an autosomal recessive lysosomal storage disorder due to the deficient activity of the acid beta-glucosidase (GCase) enzyme, resulting in the progressive lysosomal accumulation of glucosylceramide (GlcCer) and its deacylated derivate, glucosylsphingosine (GlcSph). GCase is encoded by the *GBA1* gene, located on chromosome 1q21 16 kb upstream from a highly homologous pseudogene. To date, more than 400 *GBA1* pathogenic variants have been reported, many of them derived from recombination events between the gene and the pseudogene. In the last years, the increased access to new technologies has led to an exponential growth in the number of diagnostic laboratories offering GD testing. However, both biochemical and genetic diagnosis of GD are challenging and to date no specific evidence-based guidelines for the laboratory diagnosis of GD have been published. The objective of the guidelines presented here is to provide evidence-based recommendations for the technical implementation and interpretation of biochemical and genetic testing for the diagnosis of GD to ensure a timely and accurate diagnosis for patients with GD worldwide. The guidelines have been developed by members of the Diagnostic Working group of the International Working Group of Gaucher Disease (IWGGD), a non-profit network established to promote clinical and basic research into GD for the ultimate purpose of improving the lives of patients with this disease. One of the goals of the IWGGD is to support equitable access to diagnosis of GD and to standardize procedures to ensure an accurate diagnosis. Therefore, a guideline development group consisting of biochemists and geneticists working in the field of GD diagnosis was established and a list of topics to be discussed was selected. In these guidelines, twenty recommendations are provided based on information gathered through a systematic review of the literature and two different diagnostic algorithms are presented, considering the geographical differences in the access to diagnostic services. Besides, several gaps in the current diagnostic workflow were identified and actions to fulfill them were taken within the IWGGD. We believe that the implementation of recommendations provided in these guidelines will promote an equitable, timely and accurate diagnosis for patients with GD worldwide.

## Background

Gaucher disease (GD- OMIM #230800) is an autosomal recessive lysosomal storage disorder due to the deficient activity of the lysosomal hydrolase, acid beta-glucosidase (GCase; EC 3.2.1.45). The enzyme is present in the lysosomes of all nucleated cells and cleaves the beta-glucosidic linkage of glucosylceramide (GlcCer) yielding glucose and ceramide. Therefore, the deficiency of GCase leads to the progressive lysosomal accumulation of GlcCer and its deacylated derivate, glucosylsphingosine (GlcSph) mainly in the monocyte/macrophage system, resulting in multiorgan dysfunction [1].

The disease presents as a continuum of phenotypes, ranging from severe forms presenting at birth to very mild phenotypes. However, in the words of Knudson, broadly speaking, three main forms of the disease can be recognized: Type 1, non-cerebral; Type 2, cerebral acute; Type 3 cerebral chronic [2].

Type 1 GD (MIM No. 230800), the most common phenotype, is characterized by enlargement and dysfunction of the liver and spleen, displacement of normal bone marrow by storage cells and bone damage leading to infarctions and fractures. Although type 1 GD is considered a non-neuronopathic form, there is increasing evidence of neurological involvement in these patients (ie Parkinson syndrome, and Lewy body dementia [3–7]. Type 2 GD (MIM No. 230900) is a rare phenotype associated with an acute neurodegenerative course and death at a very early age; while type 3, the chronic neuronopathic GD (MIM No. 231000), comprises an extremely heterogeneous group of patients who present with either attenuated or severe systemic disease associated with neurological involvement originating in childhood to early adulthood [8, 9].

The human GCase is encoded by the *GBA1* gene (GRCh37/hg19 Chromosome 1: 155,204,239 to 155,214,653), located on chromosome 1q21. The *GBA1* gene is approximately 7.5-kb long and contains 11 exons. A highly homologous 5.5 kb-pseudogene (*GBAP*; MIM No. 606463; GenBank accession no. J03060.1) has been located 16 kb downstream from the active gene [10].

GCase protein is synthesized on polyribosomes as a 55-kDa peptide, which is then translocated into the endoplasmic reticulum (ER), where it is modified by the addition of high mannose oligosaccharides and transported to the trans-Golgi network from where it is trafficked to the lysosomes [11]. GCase protein is targeted to the lysosomal compartment through a mannose 6-phosphate-independent receptor, the lysosomal integral membrane protein type 2 (LIMP-2) [12], a trans-membrane protein mainly found in the lysosomes and late endosomes [13, 14]. At the acidic lysosomal pH, LIMP-2 dissociates from GCase enabling enzymatic activity facilitated by the co-factor Saposin C (Sap C) [15–18].

The diagnosis of GD is based on the demonstration of deficient GCase activity in cells and the identification of pathogenic variants in the *GBA1* gene.

Latterly, the development of new technologies has improved the diagnostic capacity of expert laboratories. At the same time, increased access to these technologies has led to an exponential growth in the number of diagnostic laboratories that offer GD testing. However, both biochemical and genetic diagnosis of GD are challenging and to date no specific, evidence-based guidelines for the laboratory diagnosis of GD have been published.

The objective of the guidelines presented here is to provide evidence-based recommendations for the technical implementation and interpretation of biochemical and genetic testing for the diagnosis of GD to ensure a timely and accurate diagnosis for patients with GD worldwide.

## Methods

The guidelines have been developed by members of the Diagnostic Working group of the International Working Group of Gaucher Disease (IWGGD), a non-profit network established to promote clinical and basic research into GD for the ultimate purpose of improving the lives of patients with this disease.

One of the goals of the IWGGD is to support the provision of equitable access to diagnostic testing and the introduction of standardized procedures that ensure patients with GD can readily obtain an accurate diagnosis.

A guideline development group (GDG) consisting of biochemists and geneticists working in the field of GD diagnosis was therefore established and a list of guideline topics were selected for development.

A systematic literature review on GD biomarkers, biochemical diagnosis, GCase activity, molecular diagnosis and *GBA1* mutations was carried out using Medline and the Cochrane Library. The literature search on molecular diagnosis and *GBA1* mutations was limited to the last 20 years. The following search terms were used: “Gaucher” and “biomarkers” or “chitotriosidase or CCL18 or PARC or glucosylsphingosine, lysoGL1 or lysoGb1 or ACE or angiotensin converting enzyme or tartrate resistant acid phosphatase or TRAP or tartrate-resistant acid phosphatase”; “Gaucher” and “activity” and “fibroblasts or leukocytes” and “sensitivity or specificity or predictive value or analytical range”; “Gaucher” and “dry blood spot or dried blood spot or DBS”; “Gaucher” and “NGS”; “*GBA* or *GBA1*” and “NGS or Sanger”; “Lysosomal storage disorders” and “NGS”; “Gaucher” and “frequency and mutation”; “Gaucher” and “genotype” and “registry”. Searches were limited to English language publications only.

One hundred eighty-six papers were selected as relevant.

References related to a single topic (i.e., biomarkers, enzyme activity, genetic testing) were pulled together and the GDG was divided into subgroups to critically revise references, grade them, write a draft summarizing evidence and formulate recommendations.

The group met three times virtually (December 10th, 2020; July 12th, 2021; December 21, 2021) and corresponded by email regularly for the duration of the guideline development.

All GDG members discussed the draft documents. Evidence levels were classified in accordance with the method proposed by Burns et al. [19] (Tables 1, 2).

**Table 1:** Level Type of evidence

Level	Type of evidence
I	High quality prospective cohort study with adequate power or systematic review of these studies
II	Lesser quality prospective cohort, retrospective cohort study, untreated controls from an RCT, or systematic review of these studies
III	Case–control study or systematic review of these studies
IV	Case series
V	Expert opinion; case report or clinical example; or evidence based on physiology, bench research or “first principles”

**Table 2:** Grade of recommendation & criteria

Grade	Descriptor	Qualifying Evidence
A	Strong recommendation	Level I evidence or consistent findings from multiple studies of levels II, III, or IV
B	Recommendation	Levels II, III, or IV evidence and findings are generally consistent
C	Option	Levels II, III, or IV evidence, but findings are inconsistent
D	Option	Level V evidence: little or no systematic empirical evidence

These guidelines will be revised every 2 years to update the recommendations in light of the development and validation of novel diagnostic methods.

**Topics**:

1. Biomarkers of GD assisting in diagnosis
  A. Biomarkers described in GD:
    Chitotriosidase activity
    PARC/CCL18 (pulmonary and activation-regulated chemokine)
    Glucosylsphingosine (GlcSph, lysoGL1, lysoGb1).
    ACE (angiotensin-converting enzyme)
    TRAP (tartrate-resistant acid phosphatase)
    gpNMB (glycoprotein nonmetastatic melanoma protein B)
  B. Biological materials and methods used to assess recommended biomarkers
2. Enzyme activity
  A. In what samples glucocerebrosidase (GCase) activity can be measured?
  B. How GCase activity can be measured?
  C. What is the role of enzymatic activity in GD diagnosis?
  D. How to validate GCase assay in the laboratory?
3. Genetic testing
  A. What is the role of genetic testing in the diagnosis of GD?
  B. How should molecular testing be performed?
  C. Conditions with a biochemical profile suggestive of GD and no pathogenetic variants in *GBA1* gene
4. Use of Dried Blood Spot (DBS) samples for diagnosis in external laboratories
5. Final conclusions and algorithms
6. Future challenges

## Biomarkers of Gaucher disease assisting in diagnosis

Biomarkers are in general chemical entities, ranging from simple metabolites to complex proteins, which indicate the presence of a biological process linked to the clinical manifestations and outcome of a particular disease. They are the focus of much research and, when available, they play a critical role in the diagnosis, monitoring of disease progression as well as the assessment of therapeutic interventions.

An ideal biomarker should fulfill a number of criteria.

For diagnostic purposes it should be significantly elevated in the disease with no overlap in the values obtained in untreated patients and quantification in healthy subjects. The analyte should not be influenced by factors that are unrelated to the disease. It should change in response to specific treatment. Finally, reliable, fast, and cheap methods should be available for its estimation in easily accessible biological materials (For FDA view on Biomarkers see: https://www.fda.gov/about-fda/innovation-fda/fda-facts-biomarkers-and-surrogate-endpoints).

Distinct biomarkers of GD can be recognized. The first category is associated with the presence of Gaucher cells (e.g. Chitotriosidase and CCL18/PARC) while the second includes the lipid, glucosylsphingosine (GlcSph)- sometimes termed ‘lysoGL1’ or ‘lysoGb1’ in literature supported by different companies- which accumulates as a result of the deficiency of GCase activity in cells.

### Biomarkers described in Gaucher diseases

*Chitotriosidase* Chitotriosidase is the human analogue of chitinases from lower organisms; the enzyme is released from pathological macrophages in Gaucher disease.

Sensitivity: In terms of diagnosing GD, assaying plasma chitotriosidase activity is commonly employed in many centers as a first line screening test. The activity of chitotriosidase in plasma is elevated up to 1000-fold above the mean values in a healthy reference population. In the initial studies of chitotriosidase, plasma activity was found to be elevated on average 641-fold (median control plasma, 20 nmol/mL/h; range, 4–76 nmol/mL/h; median GD plasma, 12 824 nmol/mL/h; range, 3122–65 349 nmol/mL/h) [20]. Several subsequent reports have confirmed these findings [21–24]. Generally, higher plasma chitotriosidase activity is observed in type 1 patients than patients with types 2 and 3. Increased activity has also been reported in asymptomatic/ pre-symptomatic patients identified through the screening of family members of an index case [25].

The interpretation of plasma chitotriosidase activity is complicated by the occurrence of an intragenic 24-base pair (bp) duplication in the chitotriosidase gene *CHIT1*, which prevents the formation of chitotriosidase protein. This effectively null allele is frequent in most populations, and among GD patients, where one in every three individuals is a heterozygous carrier and about one in every 20 individuals is homozygous for the mutation [26]. Several other mutations which affect chitotriosidase activity, have been described [27–31].

Specificity: Increased plasma chitotriosidase activity is not unique to GD patients. Modest elevation of activity is also found in many different lysosomal and non-lysosomal diseases such as Niemann-Pickdisease type C, Acid sphingomyelinase deficiency, Alagille syndrome, Amyotropic lateral sclerosis, hydrops fetalis due to congenital herpes virus infection, neonatal systemic candidiasis, sarcoidosis, leprosy, arthritis, multiple sclerosis, thalassemia, chronic obstructive pulmonary disease (COPD), malaria, and atherosclerosis. Generally, although the levels of activity detected in these disorders may be within the range observed in GD (especially those patients receiving specific therapy), the values are lower than those found in GD patients. Indeed, in the absence of the intragenic duplication in *CHIT1*, a marked elevation of chitotriosidase activity in plasma appears to be characteristic of and diagnostic for GD [20, 32–41]. Individuals who are homozygous for this CHIT1 allele have effectively no or near-absent chitotriosidase activity.

**PARC/CCL18:** pulmonary and activation-regulated chemokine (PARC, systematic name CCL18), a member of the C–C chemokine family which like chitotriosidase, accumulates in the alternatively activated macrophages that accumulate in GD “Gaucher cells” [42] but appears to be actively released.

Sensitivity: A 10- to 50- fold increase in the abundance of PARC/CCL18 has been reported in plasma and serum of symptomatic GD patients compared with healthy individuals [23, 24, 43]. Increased PARC/CCL18 polypeptides release s has been reported in the asymptomatic identical twin of a patient with severe disease which were substantially lower than in the symptomatic patient. PARC/CCL18 is stable upon storage and multiple freeze thaw cycles.

Specificity: Increased concentrations that can overlap those found in GD have been described in patients with a-mannosidosis and Niemann-Pick disease type A and B [23, 34]. Non-lysosomal storage diseases with increased PARC/CCL18 levels include atherosclerosis, rheumatoid arthritis, beta-thalassemia, sarcoidosis [36, 44–46]. So far, no genetic variations that significantly alter the concentrations of PARC/CCL18 have been described. Of note, PARC/CCL18 chemokine is not expressed in mice.

### *G*lucosylsphingosine (a.k.a. lysoGL1, lysoGb1)

Sensitivity: an average 180—fold increase in the concentration of GlcSph has been reported in plasma and serum of symptomatic type 1 GD patients compared with healthy individuals [47, 48]. A similar abnormality is noted in mice and zebrafish with deficient GCase [49–51]. This characteristic abnormality has been confirmed by numerous laboratories worldwide (e.g. [52, 53]; recently reviewed in [54, 55]).

*S*pecificity: More modestly increased levels of plasma GlcSph have also been noted in patients suffering from Action Myoclonus Renal Failure syndrome with a defective LIMP-2 [56], patients with Sap C deficiency [57] and in some patients with Niemann-Pick disease type C [58].

**ACE (angiotensin-converting enzyme):** A 2–tenfold increase in ACE has been described in serum/plasma of GD patients apparently originating from storage cells [23, 59–65]. Increased serum/plasma ACE has been reported in other disorders involving activation of the monocyte/macrophage lineage and sarcoidosis is the most frequent and the better studied [66]. Increased activity is not observed in all GD patients [60] up to fivefold variation in blood ACE across a population can be observed and several mutations/polymorphisms in the ACE gene have been described which result in increased ACE blood levels [67, 68]. ACE activity can be repressed in patients who are take ACE inhibitors [69].

**TRAP (tartrate-resistant acid phosphatase):** TRAP was the first biomarker to be assayed in the diagnosis of GD [70]. TRAP is not specific for GD and the observed increase in the serum is modest. It is unstable in the blood and shows marked analytical variability [23, 62]. In interpreting TRAP serum levels, its increased activity in children as compared to adults should be taken into consideration together with its thermo-instability [71].

**gpNMB (glycoprotein nonmetastatic melanoma protein B):** gpNMB has been identified by proteomics analysis of laser dissected Gaucher cells from GD spleens [42, 65, 72]. It is selectively overexpressed by Gaucher cells that release a soluble fragment into plasma that can be conveniently detected by ELISA. The soluble fragment of gpNMB is found to be elevated over 50-fold in plasma of patients with type 1 GD [72] and was also found to be elevated in human NPC plasma samples [65]. A recent investigation confirms the value of soluble gpNMB as a plasma marker of Gaucher cells and substantiates its diagnostic potential [73]. However further studies are needed before its role as a diagnostic biomarker is established.

- **Recommendation #1:** Based on the data available to date, it is recommended that chitotriosidase activity, PARC/CCL18 or GlcSph concentrations can be used as a first line test when the diagnosis of GD is suspected.
- If chitotriosidase activity is the only assessed biomarker and the result is normal, the presence of the 24 bp duplication in the CHIT1should be excluded. In these cases, measurement of the PARC/CCL18 and/or GlcSph is recommended.
- However, a suspected diagnosis of GD needs to be established by assay of GCase activity (in peripheral blood leukocytes or extracts of cultured fibroblasts), preferably supported by molecular analysis of the GBA1 gene or by the identification of biallelic pathogenetic variants in the GBA1 gene
- **Level of evidence**: II (cohort studies /case series with consistent results/ research articles)
- **Grade**: B (Recommendation)

### Biological materials and methods used to assess recommended biomarkers

Assaying chitotriosidase activity: The biological material to be used is serum and/or plasma. The enzyme in plasma is stable upon storage and multiple freeze thaw cycles, (storage: stable at room temperature for 24 h; storage at -30 after 8 months recovery 95.3–102%, data presented by Aerts et al. GD Biomarker Qualification Workshop, September 2010, FDA Campus). Although the use of DBS in the diagnosis of lysosomal storage disorders has become increasingly popular mainly due to its convenience, at present extensive studies documenting sensitivity and specificity of assaying chitotriosidase activity in this type of biological material are not yet available [74–77]. The activity of chitotriosidase in plasma/serum can be determined using the fluorogenic substrate 4- methylumbelliferyl-β-D-N,N′,N″-triacetyl-chitotrioside (4MU-C3).However, the assay is complicated by the ability of chitotriosidase to transglycosylate as well as hydrolyze this substrate and thus the reaction has nonlinear kinetics with respect to time shows non-Michaelis–Menten behaviour [78]. Therefore, it is essential that special care is taken to ensure that the enzyme activity is truly proportional to the amount of chitotriosidase protein and there is an urgent need to standardize the assay across laboratories.. Alternatively, a far more convenient, sensitive, and accurate detection can be achieved by measuring the activity of chitotriosidase toward the fluorogenic substrate 4-methylumbelliferyl-deoxychitobiose (4MU-dC2). Chitotriosidase shows normal Michaelis–Menten kinetics with this substrate, allowing the use of saturating substrate concentrations. Thus, a more accurate and robust assay is now available [78, 79].

Measurement of the levels of PARC/CCL18: The biological material to be used is serum and/or plasma. PARC/CCL18 is stable upon storage and multiple freeze thaw cycles (storage: stable at room temperature for 48 h; storage at-30; 8 month recovery 107–109%, data presented by Aerts et al. GD Biomarker Qualification Workshop, September 2010, FDA Campus). Its levels cannot be reliably estimated using SELDI-TOF but enzyme-linked immunosorbent assay (ELISA) and dissociation-enhanced lanthanide fluoroimmunoassay (DELFIA) can be used for reliable estimation [24, 43, 80].

Measurement of the levels of GlcSph: Different techniques can be used for detection of which LC–MS/MS is presently the most sensitive. Its levels cannot be reliably estimated using SELDI-TOF. Reliable determination of absolute concentrations of GlcSph by mass spectrometry requires use of an appropriate internal standard. The concentration of GlcSph can be measured in either plasma or serum. GlcSph can be quantified in previously frozen serum or plasma samples.

GlcSph has been reported to be also increased in DBS of GD patients [74, 81, 82]. However, the outcome of extensive studies documenting specificity, stability and the impact of sample storage and shipping conditions on sensitivity of this biomarker in DBS is not yet available.

- **Recommendation #2**: The biological material to be used for assessment of recommended biomarkers is serum and/or plasma. Monocentric studies report good sensitivity of DBS GlcSph assessment in identifying GD patients. However, the outcome of extensive studies documenting specificity, stability and the impact of sample storage and shipping conditions on sensitivity of this biomarker in DBS is not yet available.
- **Recommendation #3:** Chitotriosidase can be measured using fluorogenic substrates: 4-methylumbelliferyl-β-D-N,N′,N″-triacetyl-chitotrioside (4MU-C3) or 4-methylumbelliferyl-deoxychitobiose (4MU-dC2), which allows a more convenient, sensitive, and accurate measurement of activity. If 4MU-C3 is used it is important to ensure that the enzyme activity is truly proportional to the amount of chitotriosidase protein and the need to standardize the assay across laboratories is urgent and is underway through the IWGGD Biomarkers & Materials working group.
- **Recommendation #4:** Both enzyme-linked immunosorbent assay (ELISA) and dissociation-enhanced lanthanide fluoroimmunoassay (DELFIA) can be used for reliable estimation of PARC/CCL18 concentrations, while these cannot be reliably estimated using SELDI-TOF.
- **Recommendation #5:** The most sensitive technique to assess GlcSph is LC–MS/MS. Reliable determination of absolute concentrations of GlcSph by mass spectrometry requires use of an appropriate internal standard. Its levels cannot be reliably estimated using SELDI-TOF.
- **Level of evidence**: II (cohort studies /case series with consistent results/ research articles)
- **Grade**: B (Recommendation)

## Enzyme activity

The metabolic defect in Gaucher disease (GD) is an inherited deficiency of lysosomal membrane associated acid β-glucocerebrosidase (GCase) [83]. The basic function of GCase is degradation of the glycosphingolipid glucosylceramide (GlcCer), also known as glucocerebroside within acid pH to ceramide and glucose [84]. The gold standard for GD diagnosis is the demonstration of deficient GCase activity measured in peripheral blood leukocytes and/or cultured skin fibroblasts homogenates. Traditionally enzyme activity was measured by using the natural substrate glucocerebroside [85]. Nowadays, enzyme assay is carried out by the use of an artificial substrate 4-MU- β-D-glucoside. For this reason, and to avoid misinterpretation, enzyme activity assayed by the use of artificial substrate will be called BGLU.

### In what samples BGLU activity can be measured?

The BGLU activity could be measured in different samples such as DBS, leukocytes, fibroblasts and in case of prenatal diagnosis in chorionic villi sampling (CVS) or cultured amniocytes [86]. BGLU could be measured in DBS samples as a first-line laboratory test. Pre-analytical requirements are critical for reliable BGLU results from DBS samples. DBS can be obtained by application of 50–75 μL drops of blood obtained by venipuncture into heparin tubes and spotted on the Whatman®903 or S&S903 filter paper. Another option is application of the same amount of blood after finger prick on filter paper collection device onto printed circles [87, 88]. DBS should be dried for 4 h at room temperature avoiding direct illumination, and then packed in a sealed plastic bag with desiccant, and stored at 4 °C until analysis [89]. Exposure of DBS to both heat and humidity can destroy enzyme functions rapidly. Moreover, an incomplete mixed blood before spotting can result in significant variation on enzyme activity [90].

The use of DBS as first line laboratory test offers many advantages over leukocytes or fibroblasts samples including easy collection methodologies, need of a small amount of blood, and simpler transportation as samples can be shipped via regular mail at room temperature. If the DBS sample is treated appropriately, the BGLU remain stable at least for 21 days [91–93]. DBS has limitations for measurement of BGLU activity. The volume of blood applied, hematocrit, recent blood transfusions and other preanalytical steps such as drying time, homogeneity and extraction of the analyte influences the quality of the DBS sample [94]. To ensure integrity of BGLU activity and to avoid false positive results, another lysosomal enzyme should be measured as a control enzyme with approximately same stability at room temperature. The value of the control (reference) sample should generally lie between the mean ± twostandard deviations [95].

Different studies have shown good sensitivity and specificity, above 95%. However, enzyme testing in DBS has a low positive predictive value (of < 45% on average) [96–104].

Patient leukocytes or cultured skin fibroblast homogenates are the gold standard for measurement of BGLU activity. Leukocytes as the BGLU source are obtained by separation from approximately 5–10 ml of blood, drawn from the patient in potassium EDTA or heparin tubes. Moreover, skin fibroblasts should be used when patients have received blood transfusions or when discordant results are obtained with white blood cells. The shipment of blood samples to the reference laboratory should be carried out at 4 °C [105]. The isolation of leukocytes from the whole blood should be completed within 24 h after blood collection using dextran sedimentation or the ammonium chloride lysis method [106–109]. The pellet of isolated leukocytes can be stored for at least 20 days at − 20 °C before enzyme activities are determined [108].

Homogenates prepared from cultured fibroblasts are labour intensive, since they require a skin biopsy (requiring no more than local anesthesia) transport in particular medium followed by transfer to medium for a primary cell culture of skin fibroblasts (avoiding the risk of contamination). The time taken for adequate fibroblast outgrowth to obtain a confluent cell monolayer varies but is generally about three weeks. Shipment of cultured fibroblasts should be at ambient room temperature, avoiding freezing, in a tube, dish or sealed flask (T25 or T75) containing culture media [110]. There are some potential interfering factors in the assays: excessive transport time, lack of viable cells, bacterial or mycoplasma contamination, exposure of the specimen to temperature extremes (freezing or > 30 °C).

The use of gold standard samples requires a homogenisation step with a metal tip sonicator, and total protein measurement of the homogenate [105, 111].

- **Recommendation #6:** BGLU activity can be measured in dried blood spots (DBS) samples as a first-line test. However, GD diagnosis should never be rely solely on DBS enzyme activity measurement. Patient leukocytes or cultured skin fibroblast homogenates are the gold standard for measurement of BGLU activity and confirmation of GD diagnosis; skin fibroblasts, while more laborious and expensive to obtain, have the advantage that they can be cryopreserved in liquid nitrogen almost indefinitely and if adequately aliquoted, can be used repeatedly for study
- **Level of evidence**: II, III and IV (Well-designed cohort, case–control study, case reports)
- **Grade**: B (Recommendation)

### How GCase activity can be measured?

BGLU activity can be measured using fluorometric methods, tandem mass spectrometry or by digital microfluidics platforms. Fluorometric methods are based on the artificial substrate 4-methylumbelliferyl-β-D-glucopyranoside (4-MUG). They are mostly performed in microtiter plates [112–114]. The sample is put into a reaction mixture of acidic pH, sodium deoxytaurocholate, and the fluorogenic substrate, 4-methylumbelliferyl β-D-glucopyranoside (4-MUG). Sodium deoxytaurocholate is added in order to inhibit the non-lysosomal isoenzyme BGLU activity [93, 115–118]. Fluorometric enzyme assays for BGLU onto digital microfluidic platforms have the potential for simple, rapid and high-throughput selective screening of BGLU activity [119–122]. Beside digital microfluidic fluorometry, there are other available compact digital microfluidic platforms (e.g. electro-wetting based digital microfluidics) [123].

Tandem mass spectrometry enzyme assays with (LC–MS/MS) or without (MS/MS) liquid chromatography are based on non-fluorometric synthetic substrates [124–126]. This approach may be particularly suitable for high-throughput analyses with a large number of individuals at-risk and/or for newborn screening for GD [103, 127, 128]. All three technologies (approaches) are suitable for selective screening BGLU activity [96].

- **Recommendation #7:** BGLU activity could be measured using artificial substrate with fluorometric methods, tandem mass spectrometry or by digital microfluidics platform. The fluorometric method is accepted as the gold standard assay in leukocyte/fibroblast lysates. Tandem mass spectrometry or digital microfluidics platforms are generally used for DBS samples in screening studies.
- **Level of evidence**: II, III and IV (Well-designed cohort, case–control study, case reports)
- **Grade**: B (Recommendation)

### What is the role of enzymatic activity in GD?

Enzyme determinations in DBS samples are useful screening tests in clinically suspected individuals. Samples with BGLU activity below cut-off values require confirmation by measuring BGLU activity in gold standard samples: homogenates of leukocytes or fibroblasts [92, 112]. Whenever subjects present suggestive GD symptoms they must be reassessed even in the presence of normal BGLU from DBS testing [104].

An enzyme activity result of less than 15% of normal activity in homogenates of leukocytes or fibroblasts is diagnostic of GD [129]. Residual enzyme activity does not correlate with disease severity. Enzyme testing is not suitable for identification of carriers of GD nor of saposin C deficiency [118, 130, 131]. Heterozygotes may have half-normal enzyme activity, but overlapping with activity levels of healthy controls, rendering enzymatic testing for carrier status unreliable [132–134].

- **Recommendation #8:** The DBS samples are useful as a first line test in clinically suspected individuals. Samples with BGLU activity below cut-off values always require confirmation by measuring BGLU activity in gold standard samples: homogenates of leukocytes or fibroblasts. The demonstration of deficient (below 15% of mean normal activity) BGLU enzyme activity in leukocyte and/or skin fibroblast homogenates confirms GD diagnosis.
- Residual enzyme activity does not correlate with disease severity and the test is not suitable for diagnosis of heterozygotes of GD nor of saposin C deficiency.
- **Level of evidence**: II, III and IV (Well-designed cohort, case–control study, case reports)
- **Grade**: B (Recommendation)

### How to validate GCase assay in the lab?

To ensure the quality of BGLU testing performance, each laboratory should establish its own Quality Management (QM) system according to ISO15189 and participate in both internal and external quality assessments. The internal audit program monitors operations throughout the testing process and the quality system. For quality control purposes, it is necessary to include an appropriate blank and at least one affected control and one normal control sample for each run of enzyme assays. All assays should be performed in duplicate. The cut-off range, normal range, and disease range should be established by the laboratory based on its own analysis [135]. The inter-laboratory variance of numerical enzyme activity determinations could be large [136]. Reproducibility was demonstrated by intra- (*n* = 6) and inter-assay (*n* = 10) results using threshold of %CV < 15. Therefore, quality assurance and improvement in diagnostic proficiency have become essential in this area [137]. The enzyme assay is made in house by each laboratory based on the original published methods. It implies differences in units (pmol/h/disk, μmol/h/l, μmol/h/mg protein), disease cut-off; reference range, limit of detection (LOD) and limit of quantitation (LOQ). For this reason, laboratory reports from reference labs should include an interpretation of the result that reflects the conclusion of the result as normal or deficient, possible limitations of the test, and recommendations for additional testing if applicable.

The European Research Network for Evaluation and Improvement of Screening, Diagnosis, and Treatment of Inherited Disorders of Metabolism (ERNDIM) serves as an external proficiency testing program for clinical diagnostic laboratories, providing lyophilized fibroblasts for eight lysosomal storage diseases (LSD) enzymes [138]. For laboratories testing lysosomal enzymes on DBS, the Newborn Screening Quality Assurance Program (NSQAP) at Centers for Disease Control and Prevention (CDC) provides quality control (QC) materials, proficiency testing (PT) services, and technical support in collaboration with the Newborn Screening Translation Research Initiative (NSTRI) at CDC [139, 140].

- **Recommendation 9:** Each laboratory should establish its own Quality Management (QM) system, if possible, according to ISO15189 international standards and participate in both internal and external quality assessments.
- **Level of evidence**: V (Review, expert opinion)
- **Grade**: D (Option)

## Genetic testing

GD is caused by biallelic pathogenic variants in the gene encoding the acid β glucocerebrosidase protein, *GBA1* (GRCh37/hg19 Chromosome 1: 155,204,239 to 155,214,653).

A highly homologous pseudogene, *GBAP* (96% identity), is located 16 kb downstream of the *GBA1* gene [10]. The high degree of homology, which reaches 96% in exonic regions, and the proximity between *GBA1* and *GBAP* favours the occurrence of recombination events resulting in complex gene-pseudogene rearrangements [141, 142].

The nascent GCase polypeptide is composed of 536 amino acids, including 39 that encode a signal sequence that is later cleaved after it directs the polypeptide to transit the endoplasmic reticulum. Historically, *GBA1* variants were numbered from the first residue after the cleavage of the signal peptide as amino acid number one. This legacy nomenclature is still used (herein reported between brackets and without the prefix p.), although it does not comply with contemporary nomenclature standards of the Human Genome Variation Society (HGVS).

### What is the role of genetic testing in the diagnosis of GD?

The identification of biallelic pathogenetic variants in the *GBA1* gene confirms the diagnosis of GD.

Genetic testing is performed in subjects displaying absent or low residual BGLU activity in cells to support the diagnosis and provide appropriate genetic counseling to family members.

Genetic testing can be done as a primary test for GD diagnosis. However, since many *GBA1* variants are private, the chances of finding variants of uncertain significance (VUS) are quite high [25, 143–155]. In this case, confirmation of diagnosis through the assessment of enzymatic activity in patient’s cells is mandatory.

Variants should be classified following the American College of Medical Genetics (ACMG) criteria and in the case of VUS, pathogenicity should be assessed by functional analysis.

In addition, molecular testing of known familial variants represents the most reliable method to identify GD carriers since enzymatic activity does not discriminate between carriers and normal subjects [132].

According to The Human Gene Mutation Database (HGMD-Professional 2021.1), 540 variants of the *GBA1* gene have been reported to date, although not all of them are linked to GD. Indeed, 403 of them have been associated with GD.

Diverse variants have been reported: missense and nonsense variants, splice junction variants, deletions and insertions of one or more nucleotides and complex alleles (complex rearrangements) resulting from gene conversion or gene fusion with the downstream pseudogene *GBAP*. However, missense and nonsense variants, are the most frequently identified in GD patients worldwide [156].

The frequency distribution of *GBA1* variants differs across ethnic groups. While 4 pathogenetic variants (N370S; L444P, c.84–85 insG; IVS + 1G > A) account for 90% of alleles within Ashkenazi Jews, they account only for about 50–75% of alleles in non-Jewish populations. In addition, about 10% of patients present large deletions/recombinant alleles [25, 143–155, 157–164].

- **Recommendation #10:** Molecular analysis of the GBA1 gene should always be performed when biomarker results or phenotype are at odds with the enzymology and is highly recommended in subjects with BGLU activity below normal reference intervals in cells to further support/confirm the diagnosis of GD and provide genetic counseling. Testing of familial variants and genetic counseling should be made available to all at risk family members.
- **Recommendation #11:** Genetic testing could be done as a primary test (before testing enzymatic activity). However, results should be interpreted with caution since GBA1 testing is challenging (see below), depending on the method used the detection of large deletions and/or recombinant alleles will not be possible and VUS are often identified. Therefore, confirmation of diagnosis through the assessment of enzymatic activity in patient’s cells is mandatory.
- **Recommendation #12:** Genetic testing is the most reliable method to detect heterozygous carriers and it should be made available to family members at risk of being a carrier.
- **Recommendation #13:** In all cases, molecular testing should be accompanied by a pre and post-test genetic counseling delivered by a counsellor experienced in GD to ensure informed choices.
- **Level of evidence**: II and IV (retrospective cohort studies or case series with consistent results)
- **Grade**: B (Recommendation)

### How should molecular testing be performed?

Long template specific PCR amplification of the *GBA1* gene (and not the pseudogene) followed by Sanger sequencing allows the identification of single base pair variants and most recombinant alleles leading to molecular diagnosis of GD about 95–98% of cases [25, 143–155]; however, this method fails to detect large deletions [144, 151, 165, 166].

*GBA1* gene can also be analyzed using Next-Generation Sequencing (NGS) technologies, both as a single gene or as part of targeted gene panels, Whole exome sequencing (WES) or Whole genome sequencing (WGS). In all cases, the workflow should be optimized to avoid false positive or negative results due to misalignment of reads between the gene and the pseudogene.

Strategies to specifically analyze the *GBA1* as a single gene using NGS technology have been developed [167–169]. Such NGS strategies allow the identification of single base pair variants and recombinant alleles (excluding the Recdelta55) with high specificity and sensitivity [167, 169]. Conversely, analysis of the *GBA1* gene as part of gene panels using well designed NGS strategies that consider the presence of the pseudogene, allows only the identification of point mutations, while fail to identify both large deletions and recombinant alleles due misalignment of reads with the homologous pseudogene [170–174].

However, NGS data analysis is a field in continuous and rapid evolution and new solutions to improve sensitivity and specificity are expected to be available in the near future [175].

Indeed, the use of PacBio long-read Single Molecule Real-Time (SMRT) for *GBA1* deep sequencing has recently been developed [176]. However, this technology is still not widely available in most genetic laboratories.

Multiplex ligation-probe amplification (MLPA) kits have been developed for the identification of recombinant/deleted *GBA* alleles. However, commercially available kits do not discriminate between L444P mutant and *RecNci* alleles and do not discriminate between recombination events and deletions [151, 174, 177].

- **Recommendation #14:** Sequencing analysis of GBA1 exons and intron exon boundaries should be performed as the primary molecular test. It should be performed using specific long template amplification of the GBA gene (avoiding the amplification of the pseudogene) followed by Sanger sequencing or NGS specifically designed to avoid reads misalignments. This strategy allows detecting point mutations and most recombinant alleles but is not suitable to detect large deletions.
- **Recommendation #15:** GBA1 could be included in gene panels analyzed by NGS. This technology allows the detection of point mutations, although false positive results have been reported. Therefore, point mutations detected by NGS methods should always be confirmed by Sanger sequencing. Standard workflows are not suitable for the detection of large deletions or recombinant alleles.
- **Recommendation #16:** Segregation of alleles by identifying variants in parents, should be determined.
- **Recommendation #17:** The presence of homozygous pathogenetic variants not confirmed in parents, as well as the absence of pathogenetic variants (in one or both allele) after sequencing should always be questioned and additional investigations should be performed. In particular, multiplex ligation-probe amplification (MLPA) and mRNA analysis should be done to identify possible undetected recombinant/deleted alleles or deep intronic pathogenetic variants, respectively.
- **Recommendation #18**: Variants should be classified following the ACMG criteria and in case of identification of VUS, pathogenicity should be investiagted by functional analysis.
- **Level of evidence**: II and IV (retrospective cohort studies or case series with consistent results)
- **Grade**: B (Recommendation)

### Conditions with a biochemical profile suggestive of GD and no pathogenetic variants in *GBA1* gene

Although most cases of GD are due to mutations within the *GBA1* gene, a small number of patients present mutations in the *PSAP* gene which encodes the GCase activator, saposin C (Sap C) [178–183].

Sap C is a member of a family of four small lysosomal glycoproteins (Saps A, B, C and D), all derived by proteolytic processing from a common precursor protein, prosaposin (PSAP), encoded by the *PSAP* gene (NM_001042465.3) located on chromosome 10 [184, 185].

Sap C promotes rearrangement of lipid organization in lysosomal membranes favoring substrate accessibility to GCase. Therefore, mutations in the Sap C domain of *PSAP* result in the inability of GCase to degrade GlcCer, with the consequent accumulation within the lysosomes, leading to a GD like phenotype. These patients display increased chitotriosidase activity and increased levels of GlcSph. However, the in vitro GCase activity in cells results reduced or even normal since Sap C is not needed for the hydrolysis of the artificial substrate used in the diagnostic test in vitro [179–183, 186].

- **Recommendation #19:** In the absence of pathogenetic variants in the GBA1 gene in subjects with a clinical phenotype compatible with GD, increased chitotriosidase activity, increased levels of GlcSph and normal or low BGLU activity in cells, a Sap C deficiency should be suspected and the PSAP gene analyzed.
- **Level of evidence**: V (case reports)
- **Grade**: D (Option)

## Use of DBS samples for diagnosis in external laboratories

The use of dried blood spots (DBS) in the diagnosis of lysosomal storage disorders has become increasingly popular mainly due to its convenience.

As stated above, BGLU activity and GlcSph can be measured in DBS. However, results have to be interpreted with caution since BGLU testing in DBS has a very poor positive predictive value (see enzyme activity section) and although recent monocentric studies have shown encouraging results in favor of using DBS to assess GlcSph, several points require clarification before this can be recommended. In particular, stability over time of the sample (to define storage and transport time recommendations) as well as correlation between standard and DBS assays and specificity (see biomarkers and enzyme activity sections) all need to be evaluated.

- **Recommendation # 20**: DBS can be used for diagnosis of GD in patients without access to in house testing. In these cases, DBS can be sent to external laboratories with expertise in GD. Pre-analytical requirements are critical for reliable results. Both BGLU and/or GlcSph can be assessed as a first line test in this type of sample. However, interpretation of the results needs caution.
- Therefore, diagnosis should never be relied on these tests only and they should be confirmed by demonstration of biallelic pathogenetic variants in the GBA1 gene (see genetic testing section).
- In the absence of biallelic pathogenetic variants, the assessment of BGLU activity in cells is mandatory.
- **Level of evidence**: II and IV (retrospective cohort studies or case series with consistent results)
- **Grade**: B (Recommendation)

## Final conclusions

**Fig. 1:**
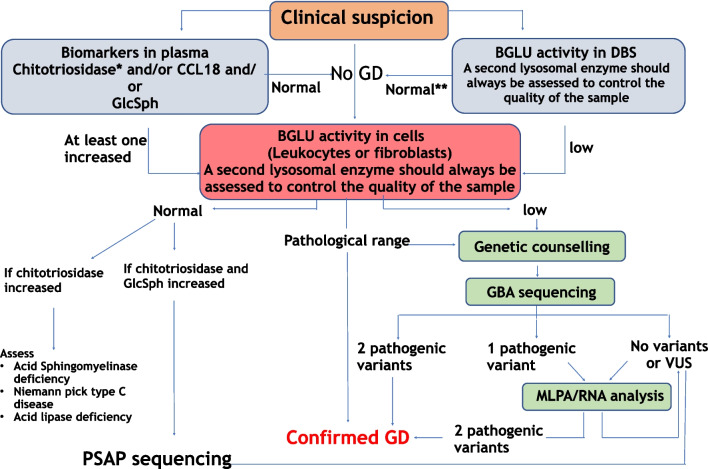
Diagnostic algorithm 1. *The presence of the 24 bp deletion has to be excluded if this is the only biomarker assessed and it results normal. **Subjects presenting suggestive GD symptoms must be reassessed even in the presence of normal BGLU in DBS

These guidelines address the laboratory workup for the diagnosis of GD type 1 and are intended to facilitate accurate and timely diagnosis regardless of demography and access to health care. Based on the gathered evidences and the recommendations above, a diagnostic algorithm has been developed as shown in Fig. 1 (Algorithm 1).

**Fig. 2:**
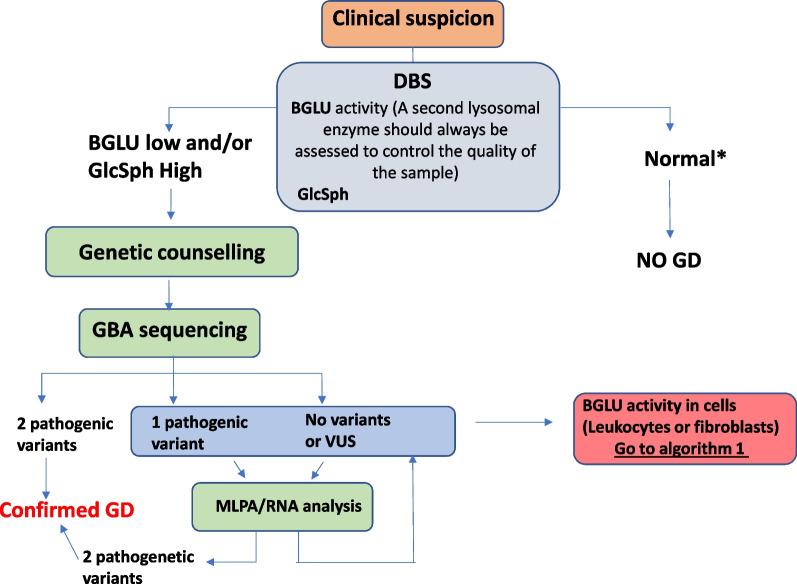
Diagnostic algorithm 2. *Subjects presenting suggestive GD symptoms must be reassessed even in the presence of normal BGLU in DBS

The group is aware that not all patients around the world have access to in-house testing and they are obliged to rely on external laboratories, sometimes commercial services, for diagnosis. In this case, dry blood spots can be used although results have to be interpreted with caution. An algorithm for diagnosis using DBS is shown in Fig. 2 (Algorithm 2):

The interpretation of the test described in this workflow can be challenging and not always straight forward. Therefore, the group recommends that expert laboratories interpret the results in the context of the clinical description of the patient. Moreover, the group strongly recommends that the report includes a clear interpretation that reflects the conclusion of the result, possible limitations of the test, and recommendations for additional testing, where applicable*.*

## Future challenges

1. A standardization of assays of various plasma biomarkers is recommended. A first step in this direction is undertaken by the IWGGD working group Biomarkers & Materials.
2. The use of DBS to assess biomarkers (e.g. GlcSph) should be confirmed by multiple centers with special attention to the influence of storage and shipment conditions.
3. The potential application of plasma biomarkers to monitor disease progression and efficacy of therapeutic intervention warrants further investigation, in consultation with other IWGGD working groups.
4. Collection of more information on plasma biomarkers in other conditions in which a (partial) deficiency of GCase activity occurs: Niemann Pick type C, Action Myoclonus Renal Failure Syndrome, Saposin C deficiency.
5. Identification of biomarkers able to predict the possible neurological involvement in newly identified patients.
6. Development of new methods for accurate and cost/effective analysis genetic testing of *GBA1.*

## Data Availability

Not applicable.

## Abbreviations

GD: Gaucher disease
GCase: Acid β-glucosidase
GlcCer: Glucosylceramide
GlcSph: Glucosylsphingosine
IWGGD: International Working Group of Gaucher Disease
ER: Endoplasmic reticulum
LIMP-2: Lysosomal integral membrane protein type 2
Sap C: Saposin C
GDG: Guideline development group
ACE: Angiotensin-converting enzyme
TRAP: Tartrate-resistant acid phosphatase
gpNMB: Glycoprotein nonmetastatic melanoma protein B
DBS: Dried Blood Spot
COPD: Chronic obstructive pulmonary disease
PARC: Pulmonary and activation-regulated chemokine
4MU-C3: 4- Methylumbelliferyl-β-D-N,N′,N″-triacetyl-chitotrioside
4MU-dC2: 4-Methylumbelliferyl-deoxychitobiose
ELISA: Enzyme-linked immunosorbent assay
DELFIA: Dissociation-enhanced lanthanide fluoroimmunoassay
CVS: Chorionic villi sampling
4-MUG: 4-Methylumbelliferyl β-D-glucopyranoside
BGLU: GCase enzymatic activity assayed with the artificial substrate in vitro
LOD: Limit of detection
LOQ: Limit of quantitation
ERNDIM: European Research Network for Evaluation and Improvement of Screening, Diagnosis, and Treatment of Inherited Disorders of Metabolism
LSD: Lysosomal storage diseases
NSQAP: Newborn Screening Quality Assurance Program
CDC: Centers for Disease Control and Prevention
QC: Quality control
PT: Proficiency testing
NSTRI: Newborn Screening Translation Research Initiative
QM: Quality Management
VUS: Variants of uncertain significance
ACMG: American College of Medical Genetics
HGMD: Human Gene Mutation Database
WES: Whole exome sequencing
WGS: Whole Genome Sequencing
NGS: Next-Generation Sequencing
SMRT: Single Molecule Real-Time
MLPA: Multiplex ligation-probe amplification
